# Factors Associated with Four or More Antenatal Care Visits and Its Decline among Pregnant Women in Tanzania between 1999 and 2010

**DOI:** 10.1371/journal.pone.0101893

**Published:** 2014-07-18

**Authors:** Shivam Gupta, Goro Yamada, Rose Mpembeni, Gasto Frumence, Jennifer A. Callaghan-Koru, Raz Stevenson, Neal Brandes, Abdullah H. Baqui

**Affiliations:** 1 Department of International Health, Johns Hopkins Bloomberg School of Public Health, Baltimore, Maryland, United States of America; 2 Muhimbili University of Health and Allied Sciences, Dar es Salaam, Tanzania; 3 United States Agency for International Development, Dar es Salaam, Tanzania; 4 United States Agency for International Development, Washington, District of Columbia, United States of America; Kenya Medical Research Institute - Wellcome Trust Research Programme, Kenya

## Abstract

In Tanzania, the coverage of four or more antenatal care (ANC 4) visits among pregnant women has declined over time. We conducted an exploratory analysis to identify factors associated with utilization of ANC 4 and ANC 4 decline among pregnant women over time. We used data from 8035 women who delivered within two years preceding Tanzania Demographic and Health Surveys conducted in 1999, 2004/05 and 2010. Multivariate logistic regression models were used to examine the association between all potential factors and utilization of ANC 4; and decline in ANC 4 over time. Factors positively associated with ANC 4 utilization were higher quality of services, testing and counseling for HIV during ANC, receiving two or more doses of SP (Sulphadoxine Pyrimethamine)/Fansidar for preventing malaria during ANC and higher educational status of the woman. Negatively associated factors were residing in a zone other than Eastern zone, never married woman, reported long distance to health facility, first ANC visit after four months of pregnancy and woman's desire to avoid pregnancy. The factors significantly associated with decline in utilization of ANC 4 were: geographic zone and age of the woman at delivery. Strategies to increase ANC 4 utilization should focus on improvement in quality of care, geographic accessibility, early ANC initiation, and services that allow women to avoid pregnancy. The interconnected nature of the Tanzanian Health System is reflected in ANC 4 decline over time where introduction of new programs might have had unintended effects on existing programs. An in-depth assessment of the recent policy change towards Focused Antenatal Care and its implementation across different geographic zones, including its effect on the perception and understanding among women and performance and counseling by health providers can help explain the decline in ANC 4.

## Introduction

Antenatal care (ANC) for pregnant women by health professionals maintains women's health during pregnancy and improves pregnancy outcomes by identifying and managing pregnancy related complications [1]–[3]. ANC visits are a platform for delivery of evidence-based clinical interventions, counseling on maternal health, birth and emergency preparedness. The World Health Organization (WHO) recommends all women with uncomplicated pregnancies to attend four ANC visits during the course of the pregnancy. During ANC, the WHO recommends that women should receive tetanus toxoid immunization, intermittent preventive treatment of malaria, deworming, iron and folic acid, and insecticide treated bednets [4]. Pregnant women can also be screened for signs associated with high probability of complications and subsequent specialized care can be arranged. For example, in HIV-endemic countries, antenatal care includes HIV testing and is an entry point for prevention of mother-to-child transmission services [4]. Antenatal care attendance is also associated with an increase in facility based deliveries and use of postnatal services [5].

Although ANC is considered an important intervention for reducing maternal and newborn mortality, and the achievement of Millennium Development Goals 4 and 5, ANC services tend to be under-utilized in low-income settings [6]–[8]. Among the 69 countries tracked by the countdown to 2015, the median coverage rate of at least one ANC visit is 88% and four or more ANC visits is 55% [9]. Furthermore, descriptive analyses from multiple Tanzania Demographic and Health Surveys (TDHS) have shown that, although coverage of at least one ANC visit is almost universal, there are growing gaps in coverage of the recommended four ANC visits with a skilled provider [10]–[12]. In 1999, among women who gave birth in five years preceding the survey, approximately 96% of the pregnant women in Tanzania attended at least one antenatal care visit from a skilled provider, and only 71% of these women had four antenatal care visits (ANC4). According to the estimates from 2004/05 and 2010 TDHS, approximately 97% and 98% of the pregnant women in Tanzania attended at least one antenatal care visit, respectively. However, the percentage of the women who had four antenatal visits (ANC 4) declined markedly to 62% and 43%, respectively. The decrease in coverage of four ANC visits represents a concern for the Ministry of Health and Social Welfare (MoHSW) and partner organizations, who would like to understand this decline and address barriers to accessing the recommended ANC services across all 8 geographic zones.

The ability to fully utilize ANC services in developing countries is affected by a number of factors. These factors include availability, accessibility, and quality of health services including ANC services and women's socio-economic status, demographic factors, education, knowledge of the importance of ANC services, cultural beliefs, and previous obstetric history [6], [13], [14]. The growth and addition of disease-specific initiatives, particularly programs focused on HIV and malaria, to existing health services have increased the complexity of provision of health services and have raised concern among some observers that these might have weakened the existing health systems and adversely affected pre-existing programs[15], [16]. It is also widely accepted that health systems are complex with components that are inter-connected and sensitive to changes anywhere in the system [17]. Therefore, it is not clear whether disease-specific preventive initiatives detract or strengthen the ability of existing health systems to deliver maternal health services like ANC.

This paper examines the trends and determinants in antenatal care utilization and reasons for decline in ANC 4 over a ten-year period in Tanzania. There is limited empirical evidence about the interaction between coverage of recommended health services like ANC, scale up of disease-specific global health initiatives and changes in existing health systems in low-income countries like Tanzania. Based on WHO recommendations the MoHSW, United Republic of Tanzania, introduced focused antenatal care (FANC) in 2002 and replaced the earlier recommendation on monthly ANC visits with four antenatal visits (ANC 4). It is important to understand if changes in health programs at national level might simultaneously increase the utilization of services in some geographic zone(s) or by certain groups, and decrease the utilization in some other geographic zone(s) or by other groups. This study, therefore, aimed to examine factors associated with; 1) utilization of four or more antenatal care visits (ANC 4) by women, and 2) decline in four or more antenatal visits by pregnant women over time in Tanzania. The results of this study will provide insights to policy makers about the different public health strategies to increase the coverage of four or more antenatal care visits among pregnant women.

## Methods

### Ethics statement

The study received ethical approval from the Muhimbili University of Health and Allied Sciences (MUHAS), Dar es Salaam, Tanzania and JHSPH Institutional Review Boards. Anonymized and de-identified data for Tanzania Demographic and Health Surveys (TDHS) conducted in 1999, 2004/05 and 2010 were obtained from the Measure DHS website [18]. The TDHS is a five-year periodic survey used to collect information from women and men aged 15–49 years about demographic and health status. Three types of questionnaires were available - the Household, Women's, and Men's questionnaire [11]. We used the data from women's questionnaire that included questions about demographic characteristics, reproductive history, pregnancy, post natal care, as well as immunization and nutrition. The sampling method of TDHS has been described in detail elsewhere [19].

A total of 25251 eligible women were interviewed for these three surveys, 4118 in 1999 [12], 10611 in 2004/05 [10] and 10522 in 2010 survey [11]. The response rates of eligible women in the 1999, 2004/05 and 2010 TDHS were 98%, 97% and 96%, respectively. In the present study, information of 8035 (1296 in 1999, 3492 in 2004/05; 3247 in 2010) women with their most recent birth within two years preceding each survey was used. This restriction was aimed to reduce recall bias of mothers about their recent pregnancies. Data on the availability of other health services, including Prevention of Mother to Child Transmission (PMTCT) of HIV and Intermittent Preventive Treatment in Pregnancy (IPTp) for Malaria were available for a woman's most recent birth in the 2004/05 and 2010 TDHS surveys only. Therefore, data from 1999 TDHS was included in descriptive analyses but excluded from regression analyses.

### Variables

The primary outcome of this study was women utilizing the recommended four or more antenatal care visits (ANC 4) from a skilled service provider [11], [20]. Antenatal care from a skilled service provider referred to any pregnancy-related services provided by skilled health personnel, such as doctor/ assistant medical officer, clinical officer, assistant clinical officer, nurse/midwife, and MCH aide (sub cadre under nurses that is being phased out). Traditional birth attendants and village health workers were not included in the definition of skilled health personnel. The analyses to identify factors associated with utilization of ANC 4 and its decline over time focused on women with their most recent birth within two years preceding each TDHS survey.

**Figure 1:**
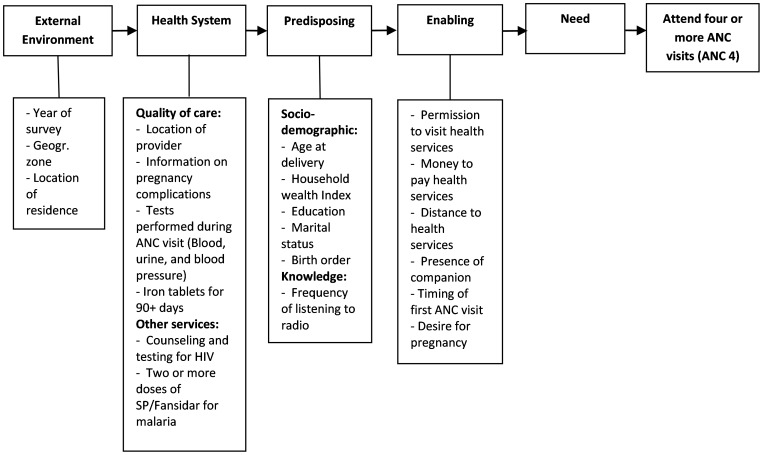
Theoretical framework of factors associated with four or more antenatal care visits (ANC 4) in Tanzania. Note: Adapted from Andersen R. M. 1995. [21]

We adapted the behavioral model for health services utilization proposed by Andersen [21] to group the factors potentially associated with ANC 4 (Figure 1). The model proposed by Andersen has been extensively used in the literature to study factors affecting health services utilization [14], [22]–[24]. The model groups factors in a hierarchical order, from proximal to distal depending on how directly the factor affects the behavior of an individual to utilize health services. Twenty three (23) potential factors were identified and categorized under four main groups: external environment, characteristics of health delivery system including quality of care, predisposing and enabling factors. These factors were selected based on studies indicating their association with ANC and availability of data within the DHS datasets. The variables representing these factors included in the study are presented in Figure 1 [21].

### Statistical analysis

Frequency tabulations were performed to analyze the distribution of factors among the women included in this study. Bivariate logistic regression analyses were conducted to assess the crude odds ratio (OR) between each of the 23 factors and utilization of four or more antenatal visits (ANC 4).

Hierarchical order of factors guided the multiple iterations of the multivariate logistic regression model [25].

The more distal factors were entered first, followed by more proximal factors to assess their associations with four or more antenatal care visits. In the first iteration, the year of survey variable and the external environment factor variables, i.e. geographic zone and location of residence were entered simultaneously (Figure 1). In the second iteration, quality of antenatal care and other services delivered were entered along with the factors associated with external environment. A similar approach was used for predisposing, and enabling factors. Change of coefficients and p-values were monitored as groups of more proximal factors were added. The year of survey was retained in all models. The final iteration were selected based on previous evidence from the literature, and whether the sub-groups had enough non-missing data to form an interpretable model (less than 10% data missing or otherwise unclassified). The interaction term between year of survey and each of the factors in the final iteration were included to identify factors associated with decline in ANC 4 over time, and only significant interaction terms at p< = 0.05 level were kept in the final model. The final model included seventeen factors and two additional interaction terms, 1) year of survey and geographic zone, and 2) year of survey and mother's age at delivery. Statistical analyses performed in this study used the STATA/IC version 12.1 [26]. Survey commands were used for all analyses to adjust for the sampling weights and cluster sampling design.

## Results

### Factors associated with utilization of four or more ANC visits

Women living in urban areas and eastern zone had higher ANC 4 rates in each TDHS round (Table 1). A higher quality of care was associated with higher rates of ANC 4, measured by variables that imply a higher rate reported among women who were informed about signs of pregnancy related complications, had their blood pressure measured and urine and blood samples taken during the antenatal care visits. The group of women who were tested and counseled on HIV prevention, and received two or more doses of Suplhadoxine-Pyrimethamine/Fansidar for prevention of malaria utilized ANC 4 at a higher rate in 2004/05 and 2010. More than 85% of the interviewed women utilized antenatal care services at public sector health facilities, with dispensaries providing ANC to more than 50%. However, within the public sector the ANC 4 rate at hospitals was higher than health centers and dispensaries.

**Table 1:** Frequencies of potential factors associated with four or more antenatal care visits (ANC 4) in Tanzania.*

	DHS 1999			DHS 2004-05			DHS 2010			Percent decline	
	Number of women	Percent ANC4 completion	(95% Confidence Interval)	Number of women	Percent ANC4 completion	(95% Confidence Interval)	Number of women	Percent ANC4 completion	(95% Confidence Interval)	Between 2004/5 – 2010	*p-value*
Four or more ANC visits (ANC 4)											
	1,296	65.0	(59.8, 69.8)	3,492	54.9	(52.3, 57.4)	3,247	37.6	(35.2, 40.1)	−31.4%	<0.001
EXTERNAL ENVIRONMENT											
*Location of residence*											
Urban	244	82.8	(77.0, 87.4)	668	63.6	(56.5, 70.2)	681	52.2	(47.5, 56.8)	−18.0%	<0.01
Rural	1,051	60.8	(54.9, 66.5)	2,824	52.8	(50.2, 55.4)	2,566	33.8	(31.2, 36.5)	−36.0%	<0.001
*Geographic Zone*											
Western	195	45.8	(39.1, 52.7)	774	37.7	(33.0, 42.7)	727	29.1	(24.9, 33.8)	−22.8%	0.01
Northern	253	62.9	(45.0, 77.9)	417	58.0	(52.4, 63.4)	392	46.2	(40.4, 52.1)	−20.3%	<0.01
Central	101	62.5	(44.5, 77.6)	295	62.5	(56.1, 68.4)	303	33.9	(26.7, 41.8)	−45.8%	<0.001
Southern Highlands	141	77.9	(59.4, 89.4)	514	54.6	(47.3, 61.8)	444	26.7	(20.5, 34.1)	−51.1%	<0.001
Lake	308	58.4	(51.8, 64.7)	781	56.0	(50.0, 61.7)	662	35.9	(30.4, 41.8)	−35.9%	<0.001
Eastern	158	88.5	(78.1, 94.3)	366	75.6	(66.7, 82.8)	393	61.0	(54.4, 67.2)	−19.4%	<0.01
Southern	107	72.1	(62.7, 80.0)	260	56.5	(49.9, 62.9)	240	37.6	(29.5, 46.4)	−33.5%	<0.001
Zanzibar	31	72.3	(62.0, 80.6)	84	65.8	(60.3, 70.9)	87	47.7	(41.4, 54.0)	−27.6%	<0.001
HEALTH SYSTEM FACTORS											
**Quality of care**											
*Location of ANC provider* †											
Public hospital	-	-	-	392	60.7	(54.0, 67.0)	286	43.9	(36.9, 51.1)	−27.6%	<0.001
Public health center	-	-	-	719	58.4	(53.6, 63.1)	693	43.0	(38.3, 47.8)	−26.4%	<0.001
Public dispensary, others	-	-	-	1,779	57.6	(54.1, 61.0)	1,823	35.9	(33.0, 38.9)	−37.6%	<0.001
Non-public (all level)	-	-	-	376	60.0	(54.0, 65.7)	294	48.6	(38.0, 59.3)	−19.0%	0.07
*Informed of signs of pregnancy complications at ANC*											
No	771	61.4	(55.3, 67.1)	1,917	51.6	(48.5, 54.7)	1,574	31.6	(28.3, 35.1)	−38.8%	<0.001
Yes	506	70.8	(65.5, 75.6)	1,552	59.0	(55.5, 62.4)	1,629	43.7	(40.7, 46.7)	−26.0%	<0.001
*Blood pressure measured at ANC*											
No	-	-		1,319	48.7	(44.9, 52.6)	1,160	28.2	(24.4, 32.3)	−42.2%	<0.001
Yes	-	-		2,167	58.5	(55.6, 61.3)	2,087	42.9	(40.1, 45.7)	−26.7%	<0.001
*Urine sample taken at ANC*											
No	-	-	-	2,151	49.2	(46.2, 52.3)	1,708	28.7	(25.7, 31.9)	−41.7%	<0.001
Yes	-	-	-	1,332	63.9	(60.3, 67.3)	1,537	47.5	(44.1, 51.0)	−25.6%	<0.001
*Blood sample taken at ANC*											
No	-	-	-	1,693	48.4	(45.0, 51.9)	811	26.4	(22.1, 31.1)	−45.6%	<0.001
Yes	-	-	-	1,794	60.9	(57.7, 64.1)	2,434	41.4	(38.7, 44.1)	−32.1%	<0.001
**Other services**											
*PMTCT/HIV: HIV pre-counseling and test done at ANC*											
Not pre-counseled and/or not tested	-	-	-	3,080	53.3	(50.7, 55.8)	1,394	29.8	(26.2, 33.8)	−44.0%	<0.001
Both pre-counseled & tested	-	-	-	340	69.1	(62.9, 74.6)	1,836	43.4	(40.6, 46.4)	−37.1%	<0.001
IPTp/malaria: Received 2+ tablets of SP/Fansidar during ANC											
Not received	-	-	-	2,733	51.2	(48.5, 53.8)	2,351	33.8	(31.0, 36.7)	−33.9%	<0.001
Received	-	-	-	722	68.8	(64.7, 72.6)	859	47.5	(43.3, 51.8)	−30.9%	<0.001
PREDISPOSING FACTORS											
**Socio-demographic**											
Age at delivery											
<20	207	67.3	(58.1, 75.3)	575	61.5	(56.6, 66.2)	466	34.7	(29.3, 40.5)	−43.7%	<0.001
20–34	894	66.1	(60.4, 71.3)	2,457	53.8	(50.8, 56.9)	2,282	39.4	(36.6, 42.3)	−26.7%	<0.001
35–49	194	57.5	(46.7, 67.6)	460	52.1	(46.7, 57.4)	499	32.2	(27.7, 37.1)	−38.1%	<0.001
*Birth order (number of children)*											
1	283	70.8	(63.3, 77.4)	754	60.1	(55.5, 64.5)	636	45.6	(40.4, 50.9)	−24.0%	<0.001
2–3	432	64.5	(57.3, 71.2)	1,241	57.2	(53.8, 60.6)	1,160	39.4	(35.7, 43.1)	−31.2%	<0.001
4–5	277	66.9	(59.6, 73.4)	746	52.4	(47.8, 57.1)	750	33.2	(28.6, 38.1)	−36.8%	<0.001
6+	304	58.5	(48.5, 67.8)	750	48.1	(43.5, 52.7)	701	32.3	(28.5, 36.5)	−32.7%	<0.001
*Marital status*											
Never married	90	61.7	(47.8, 74.0)	220	48.8	(41.1, 56.5)	223	41.4	(33.4, 49.8)	−15.2%	0.20
Currently married/living together	1,106	65.4	(60.0, 70.3)	3,022	55.0	(52.3, 57.7)	2,728	37.2	(34.7, 39.8)	−32.4%	<0.001
Formerly married	100	63.8	(51.6, 74.4)	249	58.2	(50.6, 65.4)	296	38.8	(32.1, 46.0)	−33.3%	<0.001
*Woman*'*s education*											
No education	344	53.1	(41.1, 64.9)	914	50.1	(45.9, 54.3)	828	31.2	(26.7, 36.2)	−37.7%	<0.001
Primary incomplete	219	65.5	(56.5, 73.5)	542	48.6	(43.8, 53.4)	484	29.3	(24.6, 34.4)	−39.7%	<0.001
Primary complete	684	69.5	(65.2, 73.4)	1,872	57.3	(54.2, 60.4)	1,693	39.8	(36.9, 42.8)	−30.6%	<0.001
Secondary+	49	83.2	(70.1, 91.3)	163	74.2	(65.1, 81.6)	242	61.4	(53.7, 68.6)	−17.2%	0.03
*Socio-economic status: Wealth index quintile*											
Lowest	-	-	-	789	48.2	(43.8, 52.7)	681	33.3	(29.2, 37.6)	−31.0%	<0.001
Lower	-	-	-	757	53.8	(49.1, 58.5)	774	28.1	(23.7, 33.0)	−47.8%	<0.001
Middle	-	-	-	747	52.2	(47.7, 56.7)	702	36.2	(31.7, 40.9)	−30.7%	<0.001
Higher	-	-	-	670	56.5	(51.8, 61.1)	612	42.0	(37.6, 46.6)	−25.7%	<0.001
Highest	-	-	-	528	67.9	(60.5, 74.5)	478	55.8	(49.7, 61.8)	−17.8%	0.01
*Frequency of listening to radio*											
Less than once a week	995	63.8	(58.3, 69.0)	1,447	52.0	(48.2, 55.7)	1,573	34.4	(31.0, 37.9)	−33.9%	<0.001
At least once a week	299	68.9	(61.1, 75.8)	2,040	57.0	(53.8, 60.1)	1,672	40.8	(37.8, 43.9)	−28.4%	<0.001
ENABLING FACTORS											
*Problem in getting permission to go in seeking medical care for herself*											
No problem/not a big problem	-	-	-	3,299	55.3	(52.7, 58.0)	3,143	37.8	(35.4, 40.4)	−31.6%	<0.001
Big problem	-	-	-	192	46.7	(37.5, 56.1)	99	31.4	(20.0, 45.7)	−32.6%	0.08
*Problem in getting money for treatment in seeking medical care for herself*											
No problem/not a big problem	-	-	-	2,090	56.2	(53.0, 59.4)	2,415	38.7	(35.9, 41.6)	−31.1%	<0.001
Big problem	-	-	-	1,398	52.9	(49.5, 56.3)	827	34.5	(30.6, 38.6)	−34.9%	<0.001
*Problem in distance to health facility in seeking medical care for herself*											
No problem/not a big problem	-	-	-	2,064	57.7	(54.5, 60.9)	2,512	39.2	(36.6, 41.7)	−32.2%	<0.001
Big problem	-	-	-	1,425	50.7	(46.9, 54.5)	724	32.4	(27.3, 37.9)	−36.1%	<0.001
*Problem in not wanting to go alone in seeking medical care for herself*											
No problem/not a big problem	-	-	-	2,700	56.2	(53.3, 59.1)	2,850	37.7	(35.2, 40.1)	−33.0%	<0.001
Big problem	-	-	-	788	50.4	(45.8, 55.0)	382	37.0	(30.5, 44.0)	−26.6%	<0.01
*Timing of first antenatal care visit (ANC)*											
Less than 4 months	121	94.9	(88.1, 97.9)	459	88.4	(84.2, 91.5)	433	72.3	(66.3, 77.6)	−18.1%	<0.001
4 months or higher	1,163	62.4	(57.5, 67.1)	3,030	49.8	(47.2, 52.4)	2,815	32.3	(29.9, 34.8)	−35.1%	<0.001
*Desire of last pregnancy*											
Wanted then	955	67.1	(61.4, 72.4)	2,568	56.0	(53.1, 58.9)	2,252	38.5	(35.6, 41.6)	−31.2%	<0.001
Wanted later	173	64.0	(51.7, 74.7)	709	51.8	(46.9, 56.8)	861	35.1	(31.6, 38.8)	−32.3%	<0.001
Wanted no more	168	54.0	(45.0, 62.7)	214	50.7	(42.4, 59.0)	133	38.8	(29.0, 49.6)	−23.5%	0.09

*Only women who had births in the 2 years preceding the TDHS survey interview were included.

† If woman received ANC at multiple types of health facility (<10% of total in each TDHS), the highest type was used.

In bivariate analyses, for 2004/05 women less than twenty years at the time of delivery had significantly higher odds ratio of ANC 4 as compared to women in the 20–34 year age group (Table 2). However, the odds ratio of ANC 4 for women in the 20–34 year age group at delivery was higher in TDHS 2010, though not statistically significant. Women with higher education, and higher socio-economic status also had higher odds ratio of ANC 4. Higher birth order was associated with lower odds ratio of ANC 4. Women reporting problems associated with getting permission to seek antenatal care, distance to health facilities, money, and accompanying attendants had lower odds ratio of ANC 4 in each TDHS round. Women who received their first ANC visit before 4 months of gestation had significantly higher odds ratio of ANC 4.

**Table 2:** Bivariate and multivariate odds ratios for factors associated with four or more antenatal care visits and its decline over time in Tanzania.

	Bivariate analysis (n = 6,215)			Multivariate analysis (n = 6,215)		
	*OR*	95% CI	*p*	*OR*	95% CI	*p*
EXTERNAL ENVIRONMENT FACTORS						
*Year of survey*						
2004-05	1.00	—		1.00	—	
2010	0.49	(0.42, 0.56)	<0.001	0.34	(0.28, 0.41)	<0.001
*Location of residence*						
Urban	1.00	—		1.00	—	
Rural	0.58	(0.48, 0.71)	<0.001	1.01	(0.79, 1.29)	0.94
*Geographic zone (Year 2004-05)*						
Western	0.19	(0.12, 0.30)	<0.001	0.24	(0.15, 0.37)	<0.001
Northern	0.42	(0.26, 0.69)	<0.001	0.52	(0.32, 0.82)	<0.01
Central	0.54	(0.32, 0.89)	0.02	0.71	(0.43, 1.16)	0.17
Southern Highlands	0.37	(0.22, 0.62)	<0.001	0.47	(0.29, 0.76)	<0.01
Lake	0.39	(0.24, 0.65)	<0.001	0.46	(0.28, 0.75)	<0.01
Eastern	1.00	—		1.00	—	
Southern	0.41	(0.25, 0.68)	<0.001	0.44	(0.27, 0.72)	<0.01
Zanzibar	0.60	(0.37, 0.99)	0.04	0.66	(0.41, 1.06)	0.09
*Geographic zone (Year 2010)*						
Western	0.28	(0.20, 0.40)	<0.001	0.34	(0.24, 0.49)	<0.001
Northern	0.59	(0.40, 0.87)	<0.01	0.69	(0.47, 1.02)	0.06
Central	0.34	(0.22, 0.53)	<0.001	0.46	(0.29, 0.72)	<0.001
Southern Highlands	0.25	(0.16, 0.39)	<0.001	0.31	(0.20, 0.47)	<0.001
Lake	0.38	(0.26, 0.55)	<0.001	0.48	(0.33, 0.70)	<0.001
Eastern	1.00	—		1.00	—	
Southern	0.41	(0.26, 0.64)	<0.001	0.44	(0.28, 0.69)	<0.001
Zanzibar	0.61	(0.42, 0.89)	<0.01	0.48	(0.33, 0.71)	<0.001
HEALTH SYSTEM FACTORS						
**Quality of care**						
*Location of ANC provider*						
Public hospital	1.09	(0.85, 1.40)	0.48			
Public health center	1	—				
Public dispensary, health post	0.86	(0.72, 1.02)	0.09			
Non-public	1.13	(0.85, 1.50)	0.39			
*Iron tablet/syrup taken for 90+ days during pregnancy*						
No	1.00	—				
Yes	2.42	(1.86, 3.16)	<0.001			
*Informed of signs of pregnancy complications at ANC*						
No	1.00	—		1.00	—	
Yes	1.41	(1.24, 1.60)	<0.001	1.20	(1.04, 1.38)	<0.01
*Blood pressure measured at ANC*						
No	1.00	—				
Yes	1.59	(1.37, 1.84)	<0.001			
*Urine sample taken at ANC*						
No	1.00	—				
Yes	1.82	(1.58, 2.10)	<0.001			
*Blood sample taken at ANC*						
No	1.00	—				
Yes	1.42	(1.22, 1.64)	<0.001			
**Other services**						
*PMTCT/HIV: Pre-counseled and tested for HIV at ANC*						
No	1.00	—		1.00	—	
Yes	1.04	(0.91, 1.19)	0.58	1.28	(1.07, 1.54)	<0.01
*IPTp/malaria: Took 2+ doses of SP/Fansidar during ANC*						
Not taken	1.00	–		1.00	—	
Taken	1.74	(1.51, 2.01)	<0.001	1.67	(1.44, 1.93)	<0.001
PREDISPOSING FACTORS						
**Socio-demographic**						
*Age at delivery (Year 2004-05)*						
<20 years	1.44	(1.13, 1.83)	<0.01	1.66	(1.28, 2.15)	<0.001
20–34 years	1.00	—		1.00	—	
35–49 years	0.97	(0.77, 1.23)	0.82	1.08	(0.85, 1.37)	0.53
*Age at delivery (Year 2010)*						
<20 years	0.84	(0.65, 1.10)	0.20	0.94	(0.71, 1.23)	0.64
20–34 years	1.00	—		1.00	—	
35–49 years	0.70	(0.55, 0.90)	<0.01	0.73	(0.56, 0.94)	0.02
*Number of children*						
1	1.00	—				
2–3	0.80	(0.68, 0.94)	<0.01			
4–5	0.64	(0.53, 0.78)	<0.001			
6+	0.58	(0.48, 0.70)	<0.001			
*Marital status*						
Never married	0.93	(0.73, 1.18)	0.53	0.74	(0.56, 0.99)	0.04
Currently married/living together	1.00	—		1.00	—	
Formerly married	1.04	(0.83, 1.30)	0.73	1.03	(0.81, 1.32)	0.79
*Woman*'*s education*						
No education	1.00	—		1.00	—	
Primary incomplete	0.91	(0.74, 1.10)	0.33	0.88	(0.73, 1.08)	0.22
Primary complete	1.34	(1.14, 1.57)	<0.001	1.15	(0.98, 1.36)	0.09
Secondary+	2.83	(2.09, 3.83)	<0.001	2.01	(1.45, 2.80)	<0.001
*Socio-economic status: Wealth index quintile*						
Lowest	1.00	—		1.00	—	
Lower	1.02	(0.85, 1.22)	0.85	1.02	(0.84, 1.25)	0.83
Middle	1.18	(0.97, 1.43)	0.10	1.14	(0.92, 1.41)	0.24
Higher	1.41	(1.18, 1.68)	<0.001	1.15	(0.92, 1.43)	0.22
Highest	2.32	(1.81, 2.97)	<0.001	1.20	(0.88, 1.64)	0.24
**Knowledge**						
*Frequency of listening to radio*						
Less than once a week	1.00	—		1.00	—	
At least once a week	1.30	(1.13, 1.50)	<0.001	0.95	(0.82, 1.11)	0.53
ENABLING FACTORS						
*Problem in getting permission to go in seeking medical care for herself*						
No problem/not a big problem	1.00	—		1.00	—	
Big problem	0.79	(0.57, 1.10)	0.16	0.77	(0.54, 1.10)	0.16
*Problem in getting money for treatment in seeking medical care for herself*						
No problem/not a big problem	1.00	—		1.00	—	
Big problem	0.96	(0.84, 1.09)	0.53	0.96	(0.83, 1.12)	0.64
*Problem in distance to health facility in seeking medical care for herself*						
No problem/not a big problem	1.00	—		1.00	—	
Big problem	0.89	(0.77, 1.04)	0.15	0.83	(0.70, 1.00)	0.04
*Problem in not wanting to go alone in seeking medical care for herself*						
No problem/a big problem	1.00	—		1.00	—	
Big problem	0.99	(0.84, 1.17)	0.94	1.04	(0.85, 1.26)	0.73
*Timing of first ANC visit*						
<4 months	1.00	—		1.00	—	
4+ months	0.16	(0.13, 0.20)	<0.001	0.16	(0.14, 0.18)	<0.001
*Desire of last pregnancy*						
Wanted then	1.00	—		1.00	—	
Wanted later	0.82	(0.72, 0.95)	<0.01	0.83	(0.71, 0.96)	0.01
Wanted no more	0.97	(0.73, 1.30)	0.85	0.88	(0.64, 1.21)	0.43

The multivariate analyses adjusted for all factors included in the final model (Table 2). As compared to eastern zone, every other zone (Western, Northern, Central, Southern Highlands, Lake, Southern and Zanzibar) had significantly lower odds ratio of ANC 4 (p<0.001). Health system factors significantly associated with higher odds ratio of ANC 4 were women receiving higher quality of care (information on potential pregnancy related complications) (aOR 1.20, CI 1.04–1.38), testing and counseling on HIV prevention (aOR = 1.28, CI 1.07–1.54), and women receiving two or more doses of Suplhadoxine-Pyrimethamine/Fansidar for prevention of malaria (aOR = 1.67, CI 1.44–1.93) during antenatal care visits (Table 2). Among the predisposing factors, odds ratio of ANC 4 utilization were significantly lower for never married women (aOR = 0.75, CI 0.56–0.99) and higher for women with higher educational attainment (aOR = 2.01, CI 1.45–2.80) (table 2). Women reporting distance as a problem to seek care had significantly (confidence interval includes unity) lower odds ratio of ANC 4 (aOR = 0.83, CI 0.70–1.00), and first ANC visit after four months of gestation was also associated with significantly lower odds ratio of ANC 4 (aOR = 0.16, CI 0.14–0.18). The desire to avoid the last pregnancy was associated with significantly reduced odds ratio of ANC 4 among women (aOR = 0.83, CI 0.71–0.96).

### Factors associated with decline in ANC 4 over time

Among the women who had a birth in two years preceding each TDHS survey, the percentage of women utilizing four or more antenatal care visits (ANC 4) decreased significantly from 65% (CI: 59.8–69.8) in 1999, to 54.9% (CI: 52.3–57.4) in TDHS 2004/05 (p<0.01) to 37.6% (CI: 35.2–40. 1) in TDHS 2010 (p<0.01) (Table 1). In descriptive analyses comparing TDHS 2004/05 with TDHS 2010, the rate of four or more antenatal visits (ANC 4) declined significantly across all environmental, health systems, predisposing, enabling and need factors included in this study (Table 1). However, a variable category with higher ANC 4 rate in 2004-05 was associated with a higher rate in 2010 as well. For example, between 2004-05 and 2010, the rate of decline in ANC 4 among the women who had a urine sample taken during antenatal visit was 25.6%, as compared to 41.7% decline among the women who did not have a urine sample taken. This was true for all variables included the study except geographic zone and age at delivery.

In multivariate regression, significant interaction was found between year of survey and geographic zone (p<0.01). The association between zone and ANC 4 was modified by the year of survey, i.e., when compared to the Eastern zone, each zone had a significantly different odds ratio in 2010 as compared to 2004/05. For example, as compared to Eastern zone, odds ratio of ANC 4 decreased from 0.47 (CI 0.29–0.76) to 0.31 (0.20–0.47) in Southern Highlands between 2004-05 and 2010, whereas the odds ratio of ANC 4 in Western zone increased from 0.24 (CI 0.15–0.37) to 0.34 (CI 0.24–0.49) during the same period. A significant interaction was also found between year of survey and age of women at delivery (p<0.01). As compared to the women with age between 20–34 years at delivery, women below 20 years in age and women between 35–49 years in age had a significantly different odds ratio in 2010 as compared to 2004/05. As compared to women with age between 20–34 years at delivery, higher odds ratio of ANC 4 was found among women with age below 20 years (aOR 1.66, CI 1.28–2.15) and between 35–49 years (aOR 1.08, CI 0.85–1.37) at delivery in TDHS 2004/05. The association reversed in TDHS 2010 where women with ages below 20 years (aOR 0.94, CI 0.71–1.23) and between 35–49 years (aOR 0.73, CI 0.56–0.94) at delivery had lower odds ratio of ANC 4.

## Discussion

### Factors associated with utilization of four or more antenatal care visits (ANC 4)

In this study, the factors positively associated with utilization of ANC 4 were higher quality of services indicated by the women receiving information about signs of pregnancy related complications, and higher educational status of the woman. Testing and counseling for HIV during ANC, and receiving two or more doses of SP/Fansidar for preventing malaria during ANC were also positively associated with ANC 4. The factors negatively associated with ANC 4 were residing in an zone other than Eastern zone, never married woman, reported long distance to health facility, first ANC visit after four months of pregnancy and woman's desire to avoid pregnancy. The exploration of these factors is important to develop public health strategies that address key issues that hinder women from attending four or more antenatal visits (ANC 4) in Tanzania.

**Figure 2:**
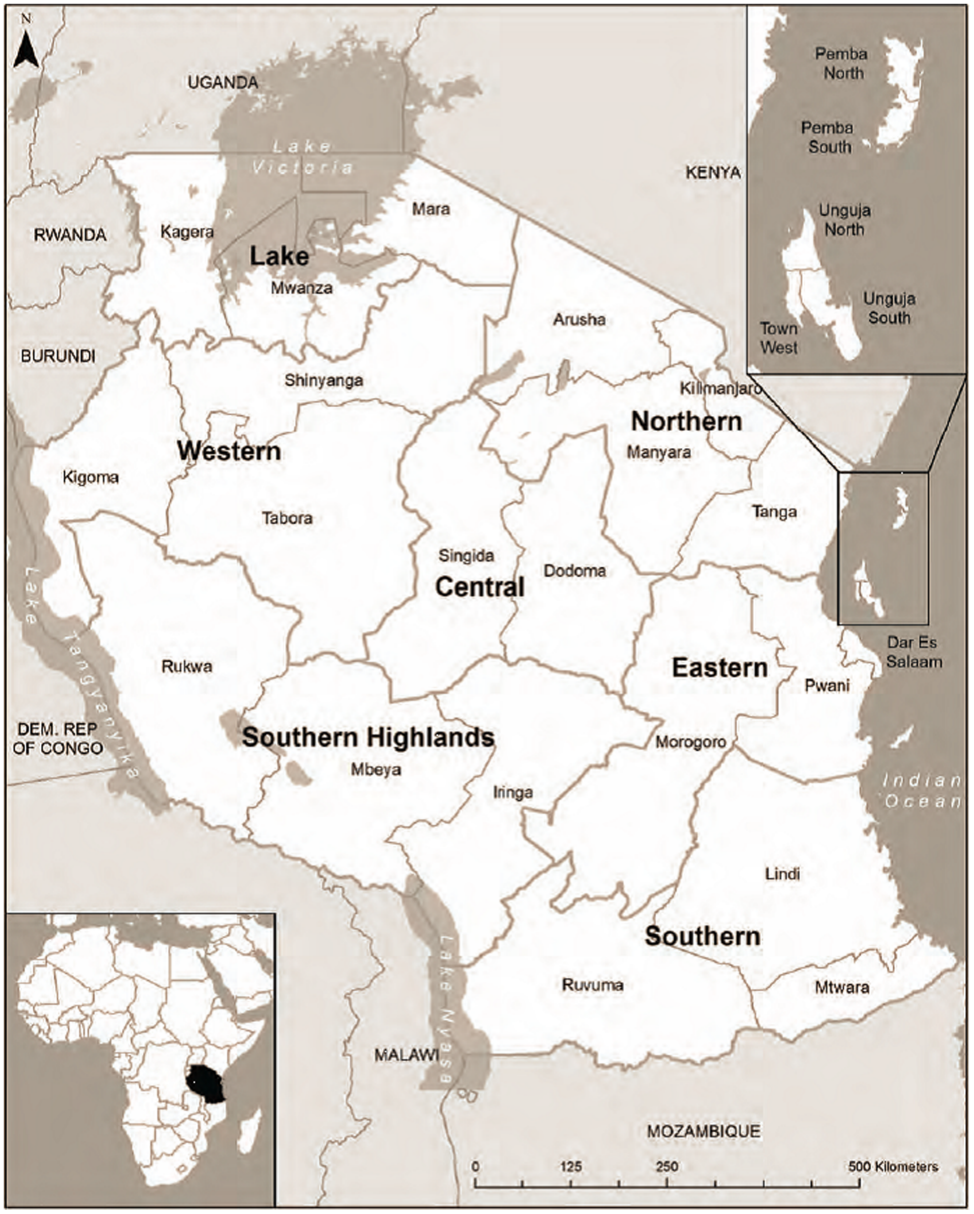
Map of Tanzania with geographic zones.

The twenty six regions in Tanzania are grouped into eight distinct geographic zones by the MoHSW and used by the TDHS surveys (Figure 2). Women residing in the Eastern zone had the highest ANC 4 utilization. All other geographic zones were associated with lower attendance of four or more ANC visits. In each TDHS, women in the Southern highlands and Western zones had the lowest utilization of ANC 4. Eastern zone includes Dar es Salaam, the country's principal commercial city, and might be associated with higher availability of good quality ANC services. A number of studies have reported the positive association between higher quality of services and higher rates of utilization of maternal health services, including antenatal care [27]–[29]. The latest study of Service Provision Assessment (SPA) within the Tanzanian Health System reports that the quality of care varies across different geographic zones. According to the SPA conducted in 2006, facilities in the Eastern zone were observed to have the highest scores on availability of testing services during ANC, and counseling for danger signs during pregnancy. Facilities in the Western zone and Southern Highlands scored among the lowest [30].

Testing and counseling for HIV during ANC, and receiving two or more doses of SP/Fansidar for preventing malaria during ANC were positively associated with ANC 4. There are several possible explanations for this association between HIV and malaria prevention programs and utilization of ANC 4. In 2004/05, 92%, and in 2010, 93% of pregnant women attended the same type of health facility in the same sector (public or private) for all their antenatal visits. During this time period, the provision of HIV and malaria prevention services at health facilities expanded in the Tanzanian health system. The percentage of women receiving counseling and testing for HIV increased from 9% to 55% between 2004/05 and 2010, with increase in availability of these services spreading from mostly hospitals in 2004/05 to addition of health center and dispensaries in 2010. The proportion of women receiving two or more doses of SP/ Fansidar during antenatal visit also increased from 22% in 2004/05 to 27% in 2010. The ANC 4 rate among women who received counseling and testing for HIV in 2004/05 and 2010 was 12.5% and 65.7%, respectively. The ANC 4 rate among women who received two or more doses of SP/ Fansidar in 2004/ 05 and 2010 was 26.2% and 33.9%, respectively. The expansion of HIV and malaria prevention services and the potential for improving quality, may have led to facility wide improvement in patient care within the health system. In most facilities, the same cadre of health worker provides ANC, HIV and malaria prevention services. HIV and malaria related increase in counseling of pregnant women by health workers and improved supervision to strengthen continuity of care might be associated with increased efforts to encourage all pregnant women to return for four antenatal visits. The association between HIV and malaria prevention programs and attendance of ANC 4 might reflect the positive dimension of global initiative funded human resource development on other health services within the Tanzanian health system [31].

High maternal education was strongly associated with four or more ANC visits. Several pathways have been suggested through which maternal education might affect health care utilization, including greater knowledge of the importance of health services among highly educated women and the increased ability to select most appropriate service for their needs [7], [32]. Higher education is also associated with increased financial and geographical access to health services.

In this present study, the problem of access to services was reflected by the decreased likelihood for ANC 4 among mothers reporting distance to health services as a major problem. Previous studies have shown that distance and time to the nearest health facilities influence health services utilization [6] [33]. A qualitative study from Zimbabwe, found that a long travel time worsened by poor road conditions prevented communities from attending antenatal services [27]. These findings indicate that the improvement of access to health services as well as the distribution of health services and personnel, especially in remote zones, should be a priority.

Other factor associated with ANC 4 found in our study included maternal desire to avoid pregnancy. Unwanted pregnancies are associated with late start or less frequent antenatal visits compared to wanted pregnancies [34]. Women with an unwanted pregnancy are more likely to underutilize antenatal services. Women may go through a period of denial and may delay the first visit in hope that pregnancy will disappear or conceal the pregnancy from friends and family [35]. Extensive literature from multiple countries indicates that the number of unwanted pregnancies can be reduced by improved availability and use of family planning services by reproductive age women [36]. In addition, after assessing wantedness of pregnancy, health providers should be trained to provide individualized, culturally-specific care and support to women with unwanted pregnancies [2].

### Factors associated with decline in ANC 4 over time

The rate of utilization for at least one antenatal care visit during pregnancy, facility based delivery and post-partum care has increased over time in Tanzania [11]. As an exception to this increasing trend in utilization of maternal health services, we found that the ANC 4 rate has declined significantly over time. At the policy level, based on the World Health Organization recommendations, The Ministry of Health and Social Welfare, United Republic of Tanzania, shifted the health system towards a new model of antenatal care in 2002 and introduced focused antenatal care (FANC), whereby the earlier recommendation on monthly antenatal care visits was replaced by each women being encouraged to attend at least four antenatal visits (ANC 4) in the course of her (uncomplicated) pregnancy [20], [37].

The decline in ANC 4 across all factors included in this study might be associated with improper implementation of FANC that coincided with the nation-wide decline in ANC 4 from 1999 till 2010. Up until the initiation of FANC in 2002, pregnant women in Tanzania were required to attend antenatal care on a monthly basis. Proper implementation of FANC requires re-training of health workers to decide the timing of four antenatal care visits at specific points during pregnancy in consultation with pregnant women based on (a) expected date of delivery; (b) presumed risk; and (c) health status [20]. Early initiation of antenatal care is a significant factor associated with a woman receiving four or more antenatal visits. In addition to promoting a total of four ANC visits, FANC strongly encourages availability of equipment to test for pregnancy and training of health workers to identify and counsel women to initiate antenatal care before four months of gestation. A number of studies have explored factors affecting delayed initiation of ANC by pregnant women. During the first four months of gestation, signs of pregnancy might not be visible externally, and a woman might not realize that she is pregnant. Even if the women realized that they were pregnant, they might not access ANC due to cultural and superstitious beliefs about pregnancy disclosure. In some cases, women might not feel the need to seek professional care when there is nothing wrong with their pregnancy [38]–[41]. A provider unclear or untrained on FANC might send a pregnant woman back home if testing facilities are not available, or if the worker concludes that it is not needed. In addition, as compared to monthly visits, the specific dates can also be more difficult to remember by the woman, especially when the reason for this scheduling is not properly explained to her.

After FANC implementation, and the subsequent reduction in maximum number of ANC visits by a woman (from monthly to four), it is reasonable to assume that system-wide, the rate of first ANC visit before four months of gestation would have increased over time. However, the proportion of women attending their first antenatal visit before four months of gestation has remained constant around 13.2% between 2004-05 and 2010. In the earlier policy on monthly visits, even a women starting after four months would have more opportunities to complete a total of four visits. Therefore, improper implementation of FANC can reduce the total number of opportunities available to women for attending a total of four or more visits. This is supported by a recent meta-synthesis of qualitative studies to explore underutilization of ANC services which hypothesized that centralized, risk-focused antenatal programs may be at odds with the resources, beliefs, and experiences of pregnant women who underuse ANC services [42]. A more in-depth assessment of FANC implementation is needed to qualitatively understand how the dates of antenatal visits are discussed and decided between the health provider and the pregnant women; what tools are available to providers to detect pregnancy and schedule visits; what information and services are provided to women who visit the facility for the first time before four months of gestation; and whether the women understand the specific dates agreed for subsequent antenatal care visits.

It is difficult to identify the specific factors associated with difference between geographic zones in rate of decline in ANC 4 over time. However, it is likely that the nationwide rollout of FANC's multiple components varied between zones, thereby affecting ANC 4 rates differentially. This might be due to variation in implementation of FANC components overlaid upon pre-existing differences in health systems performance between zones. For example, according to the SPA conducted in 2006, the quality of maternal health services was higher in the Eastern zone. Subsequently, the significant difference in rates of decline over time in zones when compared with Eastern zone might reflect the complex interplay between pre-existing health system and the expected variation in implementation of FANC related components of health worker training, equipment availability, and counseling of women over time. Our findings do provide some of the information required for initiating a much needed national debate on improving health systems performance in Tanzania [43]. However, a more in-depth and repeated assessment combining quantitative and qualitative methods focusing on the context, health provider performance and service delivery mechanisms at zonal level is needed to identify the reasons for difference in declining rates of utilizing four or more antenatal care visits between zones.

A number of studies have reported on the association between age at delivery and utilization of ANC 4 [13]. While younger women might prefer to visit often, mainly to be reassured that the baby is growing well and is in proper position, women with higher age at birth usually have high parity and might rely on their experiences from previous pregnancies and not feel the need for antenatal check-ups [27]. Some might experience difficulties to attend antenatal services due to time constraints related to their responsibilities for their other children [32]. However, some of the studies suggested that women's age was not a significant predictor of utilization of ANC [44]–[46]. In our study, as compared to 20–34 year old women, the 2010 TDHS reversal of ANC 4 utilization among both the younger (below 20 years) and older women (35–49 years) might be due to the differential impact of FANC on different age groups. A more in-depth qualitative exploration of distinctive perceptions, expectations, and resource availability of different age groups and how the health system interacted with them over time is needed to explain these changes in ANC 4.

### Strengths and limitations

This present study was based on large representative national surveys, the 1999, 2004/05 and 2010 TDHS. The potential for recall bias has been minimized by restricting the analysis only to women's most recent delivery within the last two years of each survey. The large sample used in this study allows the examination of various potential factors, the external environment, characteristics of the health delivery system, predisposing, enabling and need factors. This also increases the validity of study results. The dataset allows the examination of interconnections between a numbers of programs within the Tanzanian Health System that focus on improving the health of mothers and their newborns, for example, maternal health, HIV/ AIDS and malaria.

As with other cross sectional survey data, the interpretation of the causality of factors associated with attendance of four or more antenatal care visits is restricted by the study design. Tanzania is large and diverse country and a national level analysis cannot adjust for every contextual factor, population and health system characteristic that might affect availability and utilization of health services like antenatal care. The selection of potential factors was driven by the availability of information in each TDHS. For example, assessments of different components of health systems, including provider training and equipment availability that would be expected to change due to FANC implementation were not available for different geographic zones. However, these limitations are unlikely to impact on the validity of the analyses.
